# Conferring cellulose-degrading ability to *Yarrowia lipolytica* to facilitate a consolidated bioprocessing approach

**DOI:** 10.1186/s13068-017-0819-8

**Published:** 2017-05-19

**Authors:** Zhong-peng Guo, Sophie Duquesne, Sophie Bozonnet, Gianluca Cioci, Jean-Marc Nicaud, Alain Marty, Michael Joseph O’Donohue

**Affiliations:** 1 Biocatalysis Group, INSA/INRA UMR 792, CNRS, LISBP, Université de Toulouse, 135, Avenue de Rangueil, 31077 Toulouse, France; 2 Micalis Institute, INRA, AgroParisTech, Université Paris-Saclay, Jouy-en-Josas, France

**Keywords:** *Yarrowia lipolytica*, Cellulolytic biocatalyst, Cellulase, Endoglucanase, Cellobiohydrolase, β-Glucosidase, Consolidated bioprocessing, Cellulose

## Abstract

**Background:**

*Yarrowia lipolytica*, one of the most widely studied “nonconventional” oleaginous yeast species, is unable to grow on cellulose. Recently, we identified and overexpressed two endogenous β-glucosidases in *Y. lipolytica*, thus enabling this yeast to use cello-oligosaccharides as a carbon source for growth. Using this engineered yeast platform, we have now gone further toward building a fully cellulolytic *Y. lipolytica* for use in consolidated bioprocessing of cellulose.

**Results:**

Initially, different essential enzyme components of a cellulase cocktail (i.e,. cellobiohydrolases and endoglucanases) were individually expressed in *Y. lipolytica* in order to ascertain the viability of the strategy. Accordingly, the *Trichoderma reesei* endoglucanase I (*Tr*EG I) and II (*Tr*EG II) were secreted as active proteins in *Y. lipolytica*, with the secretion yield of EG II being twice that of EG I. Characterization of the purified His-tagged recombinant EG proteins (rh*Tr*EGs) revealed that rh*Tr*EG I displayed higher specific activity than rh*Tr*EG II on both cellotriose and insoluble cellulosic substrates, such as Avicel, β-1, 3 glucan, β-1, 4 glucan, and PASC. Similarly, cellobiohydrolases, such as *T. reesei* CBH I and II (*Tr*CBH I and II), and the CBH I from *Neurospora crassa* (*Nc*CBH I) were successfully expressed in *Y. lipolytica.* However, the yield of the expressed *Tr*CBH I was low, so work on this was not pursued. Contrastingly, rh*Nc*CBH I was not only well expressed, but also highly active on PASC and more active on Avicel (0.11 U/mg) than wild-type *Tr*CBH I (0.065 U/mg). Therefore, work was pursued using a combination of *Nc*CBH I and *Tr*CBH II. The quantification of enzyme levels in culture supernatants revealed that the use of a hybrid promoter instead of the primarily used TEF promoter procured four and eight times more *Nc*CBH I and *Tr*CBH II expressions, respectively. Finally, the coexpression of the previously described *Y. lipolytica* β-glucosidases, the CBH II, and EG I and II from *T. reesei*, and the *N. crassa* CBH I procured an engineered *Y. lipolytica* strain that was able to grow both on model cellulose substrates, such as highly crystalline Avicel, and on industrial cellulose pulp, such as that obtained using an organosolv process.

**Conclusions:**

A *Y. lipolytica* strain coexpressing six cellulolytic enzyme components has been successfully developed. In addition, the results presented show how the recombinant strain can be optimized, for example, using artificial promoters to tailor expression levels. Most significantly, this study has provided a demonstration of how the strain can grow on a sample of industrial cellulose as sole carbon source, thus revealing the feasibility of *Yarrowia*-based consolidated bioprocess for the production of fuel and chemical precursors. Further, enzyme and strain optimization, coupled to appropriate process design, will undoubtedly lead to much better performances in the future.

**Electronic supplementary material:**

The online version of this article (doi:10.1186/s13068-017-0819-8) contains supplementary material, which is available to authorized users.

## Background

The production of second-generation biofuels and platform molecules for the chemical industry from lignocellulosic biomass (LCB) is viewed as crucial part of the bioeconomy [1]. Cellulose, the main component of LCB, is a polymer composed of β-1, 4-linked glucose subunits usually embedded in an amorphous matrix of hemicellulose and lignin [2]. It may exist in two forms, a tightly packed crystalline form where individual chains are organized in microfibrils via hydrogen bonding and van der Waals interactions, or a less-ordered amorphous form [3, 4]. The depolymerization of cellulose requires a variety of enzymes, including endoglucanases (EGs) (EC 3.2.1.4) that hydrolyze internal β-glucosidic bonds, cellobiohydrolases (CBHs) (EC 3.2.1.91) that remove cello-oligosaccharides in a processive manner from chain termini, and β-glucosidases (BGLs) (EC 3.2.1.21) that degrade cello-oligosaccharides to glucose [2]. However, the condensed structure of crystalline cellulose and its intimate proximity with hemicelluloses and lignin combine to make LCB very resistant to enzymatic hydrolysis [5, 6] and render biochemical processing of raw LCB economically unviable. To overcome this recalcitrance, biomass pretreatment is necessary. However, this step constitutes a significant cost driver in the overall economics of LCB biorefinery processes [7–9]. Presently, it is widely recognized that significant technological advances, including better pretreatments, lower-cost enzymes, and efficient process design will be required in order to make LCB biorefining competitive within the current economic framework [2].

The pretreatment process operated by CIMV S.A. belongs to the so-called organosolv technology family, because it uses organic solvents to dissolve lignin and hemicelluloses, and yields a pure and relatively amorphous cellulose fraction that is quite amenable to enzyme action [10, 11]. Moreover, the CIMV process produces functionalized lignins (Biolignin^tm^) and an essentially furfural-free pentose-rich fraction, making this pretreatment technology an interesting platform for the design of a new LCB biorefinery concept.

Consolidated bioprocessing (CBP), featuring microbial enzyme production and concomitant microbial conversion of suitable feedstock into value-added products in a single step, offers great potential for cost-effective lignocellulosic bioconversion [1, 12]. This is because it is predicted that CBP will reduce both capital investment and operating costs, and possibly procure higher enzymatic hydrolysis rates [13, 14]. To date, only a few naturally occurring species of bacteria within the genus *Clostridium* have been described with such capabilities. For instance, *Clostridium lentocellum* is able to convert cellulose to acetic acid, and *Clostridium thermocellum* can ferment cellulose for the production of ethanol [15, 16]. Besides, to develop other purpose built microorganisms that will simultaneously convert cellulose pulp into sugars and ferment these to target products is still highly desired [1]. Nevertheless, using microbial engineering approaches and focusing on hosts such as *Escherichia coli* [17], *Saccharomyces cerevisiae* (reviewed in [18]) and *Kluyveromyces marxianus* [19], considerable progress has been made, although so far most studies have used model cellulose substrates and achieved relatively low titers of product. Importantly, none of the engineered strains reported so far have convincingly hydrolyzed the cellulose feedstock, and currently no commercially viable CBP organism has been reported [20].

The so-called oleaginous yeast *Yarrowia lipolytica* can accumulate lipids up to 50% of its dry weight depending on culture conditions, making this a promising platform for the production of biodiesel precursors [21, 22]. Advantageously, *Y. lipolytica* is already widely used in detergent, food, pharmaceutical, and environmental industries and has been classified by the Food and Drug Administration (FDA) as “generally recognized as safe” (GRAS) for numerous processes [23]. Moreover, *Y. lipolytica* is a suitable host for heterologous expression, since it displays high secretion ability and performs a wide-range of posttranslational modifications [24, 25]. However, regarding LCB biorefining, *Y. lipolytica* is unable to metabolize cellulose.

Recently, several reports have illustrated how cellulolytic capability can be conferred to *Y. lipolytica*, with single gene expression and coculturing being used as a pragmatic way to design a *Y. lipolytica*-based CBP system [26, 27]. However, the use of coculturing is not a feasible solution for industrial implementation, and a fully viable system can only be achieved if BGL activity is present [27]. In this respect, we recently described the overexpression of endogenous BGLs in *Y. lipolytica* and the use of cello-oligosaccharides by the recombinant yeast strain to support growth [28]. In pursuit of a more ambitious goal, in this work, we have built on this platform strain, adding other cellulolytic enzyme-encoding genes and exploring different combinations in order to procure a *Y. lipolytica* strain that is able to grow on cellulose.

## Results and discussion

### Expression of *T. reesei* endoglucanases in *Y. lipolytica*

The extensively studied cellulolytic secretome of the soft-rot fungus *T. reesei* (syn. *Hypocrea jecorina*) contains four endoglucanases, Cel7B (EG I), Cel5A (EG II), Cel12A (EG III), and Cel45A (EG V). Among these, EG I and EG II are the main endo-acting enzymes, representing approximately 15 and 10% of the total amount of cellulases (w/w) respectively, while the other two represent less than 1% each [29]. Therefore, the first step toward the construction of a fully cellulolytic *Y. lipolytica* strain was the introduction of sequences encoding *T. reesei* EG I and EG II under the control of TEF promoter and the *Y. lipolytica* lipase 2 pre–pro region into the *Y. lipolytica* zeta strain [30]. Subsequent screening of transformants producing either EG I or EG II with or without His6 revealed the presence of clones possessing the ability to hydrolyze Azo-CMC in solid agar medium (Additional file 1: Figure S1).

**Fig. 1:**
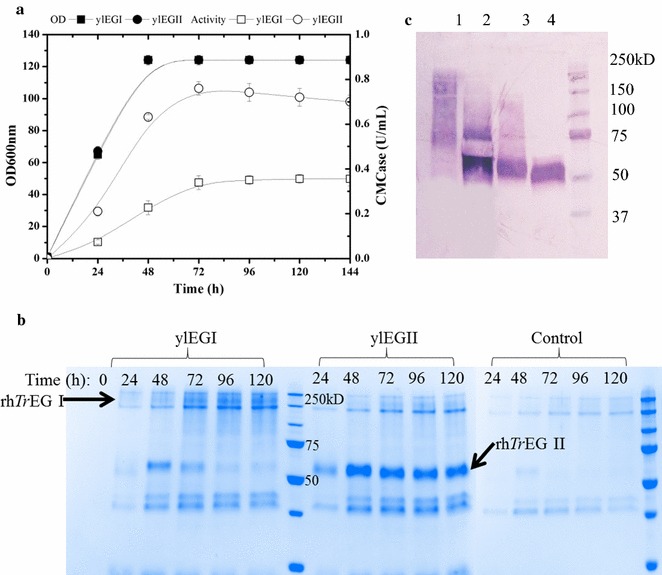
Production of rh*Tr*EG I and rh*Tr*EG II in *Y. lipolytica* **a** enzyme production on YTD versus time, **b** SDS-PAGE analysis of the culture supernatant of *Y. lipolytica* transformants compared with the control, and **c** Western blot analysis; *lanes 1* and *3*, culture supernatant of transformant ylEGI and ylEGII, respectively; *lanes 2* and *4*, culture supernatant of transformant ylEGI and ylEGII treated by endo-H, respectively

To further confirm the successful expressions of EG I and II, positive clones were grown on YTD, and the activities of EGs in culture supernatant were measured. Accordingly, CMCase activity in culture supernatant steadily increased over a 48-h period until the glucose in the culture medium was completely consumed by the yeast, with rh*Tr*EG II activity (0.78 U/mL) at the end of the period being twice that of rh*Tr*EG I (0.39 U/mL) (Fig. 1a). Furthermore, SDS-PAGE analysis of culture supernatants containing either rh*Tr*EG I or rh*Tr*EG II (Fig. 1b), compared to that of a control culture, revealed in the first case the presence of a smear (70–200 kDa) and in the latter case a discrete species migrating to a position corresponding to an approximate Mw of 55 kDa, which is a little high compared to the actual expected Mw of EG II (47 kDa). In the case of both putative rh*Tr*EG I and II, the anti-His antibody confirmed that these protein species were His-tagged (Fig. 1c). Taken together, these observations suggest that the recombinant proteins are glycosylated forms of EG I and II, in agreement with previous results [26, 29, 31] and with the findings of a bioinformatics study (http://www.imtech.res.in/raghava/glycoep/) [32] that predicted that EG I bears six potential N-glycosylation sites, while EG II possesses only one. To confirm the presence of N-glycosylation, EndoH treatment and then Western blot analysis were performed (Fig. 1c). This revealed that the EndoH-treated protein samples were still detected using anti-His antibodies, but for rh*Tr*EG I, a discrete protein species of approximately 56 kDa appeared in place of the smear, which is slightly higher than the actual expected Mw (49 kDa). Likewise, the migration of EndoH-treated rh*Tr*EG II was slightly modified, consistent with that of a lowered Mw.

### Characterization of the recombinant endoglucanases expressed in *Y. lipolytica*

**Fig. 2:**
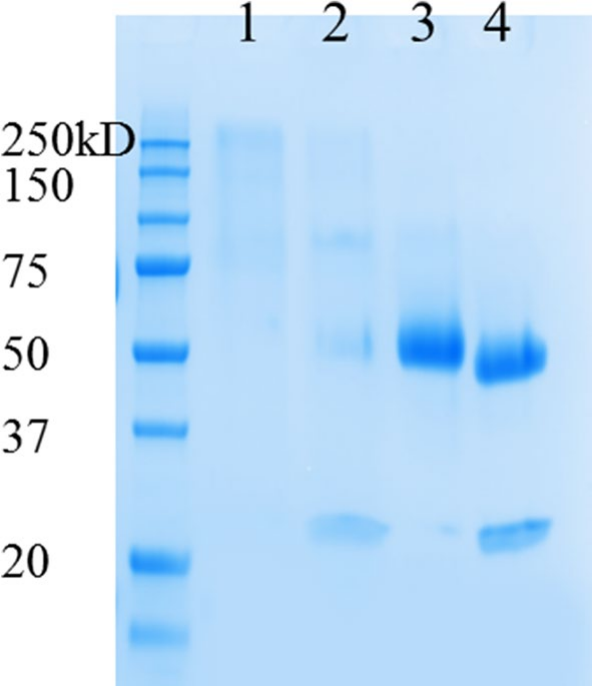
SDS-PAGE analysis of the purified rh*Tr*EG I and rh*Tr*EG II produced in *Y. lipolytica* JMY1212 transformants; *lanes 1* and *3*, purified rh*Tr*EG I and rh*Tr*EG II, respectively; *lanes 2* and *4*, endo-H treated rh*Tr*EG I and rh*Tr*EG II, respectively

Attempts to develop optimized cellulase cocktails have revealed that the efficient hydrolysis of pretreated LCB requires that EG I represents 25–35% of the total amount of cellulases (w/w) [33–35]. Moreover, such studies have underlined the usefulness of EG II for the rapid reduction of viscosity of acid pretreated wheat straw [36]. Therefore, it is important to understand the specific role of each EG in cellulose degradation in order to optimize the composition of cellulases expressed in *Y. lipolytica*. Accordingly, rh*Tr*EG I and rh*Tr*EG II were purified and characterized. For the purification of rh*Tr*EG II, a one-step affinity method procured good overall yield (>60%), whereas the yield of rh*Tr*EG I was lower (18%), probably due to the hyper-glycosylated state of this protein (Fig. 2).

Subsequently, the hydrolytic activities of purified rh*Tr*EGs were measured on various cellulosic substrates (Table 1). Significantly, compared to rh*Tr*EG II (0.1 µmol/min/mg), rh*Tr*EG I not only displayed 10 times higher specific activity on soluble cellotriose (1.1 µmol/min/mg), but also exhibited higher activity on Avicel, β-1, 3 and β-1, 4 glucans, and phosphoric acid-swollen cellulose (PASC). On the other hand, the specific activities of the two proteins on soluble carboxymethyl cellulose (CMC) were similar. Moreover, it is noteworthy that rh*Tr*EG I exhibited highest hydrolytic activity on β-1, 4 glucan, while CMC was the preferred substrate for rh*Tr*EG II. Similar substrate preferences have been reported for both the native EGs and the catalytic domains of the two proteins expressed in *E. coli* [34, 37]. However, the specific activities of rh*Tr*EG I and II expressed in *Y. lipolytica* were higher than previously reported values for the native EGs, although these differences could be linked to the quantification methods employed in each case [34, 35].

**Table 1:** Comparison of the hydrolytic activity of purified rh*Tr*EG I and rh*Tr*EG II expressed in *Y. lipolytica* on various cellulosic substrates

	Specific activity (μmol/min/mg)					
	Avicel	β-1, 4 glucan	β-1, 3 glucan	CMC	PASC	Cellotriose
EG I	0.04	18.0	0.5	12.8	9.0	1.1
EG II	0.02	11.3	0.2	11.6	7.1	0.1

The mean value of three independent experiments is shown, and the standard deviation is less than 10%

The amount of rh*Tr*EG I and rh*Tr*EG II secreted by *Y. lipolytica* in YTD medium during flask batch culture, as calculated from the total CMCase activity of the culture supernatant and specific activity of the enzyme, were approximately 29 and 67 mg/L, respectively. The secretion yield of rh*Tr*EG II obtained in this study was comparable to that reported in the literature [26, 29]. However, the amount of secreted rh*Tr*EG I was higher than the previously reported value (5 mg/L). In this respect, it is important to note that in the previous work, EG I was produced with the pre-pro region of XPR2 and under the control of the *XPR2* promoter [31]. Nevertheless, we were unable to determine why the secretion yield of rh*Tr*EG I was approximately 60% lower than that of rh*Tr*EG II. The identification of key factors that affect protein expression levels will be important for future use of *Y. lipolytica* as an efficient expression host.

### Expression of cellobiohydrolases in *Y. lipolytica*

**Fig. 3:**
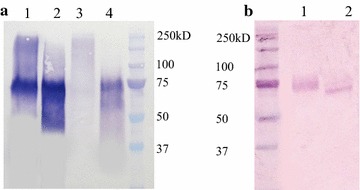
Western blot analysis of the heterologous CBH proteins produced by *Y. lipolytica* **a** *lanes 1* and *3*, rh*Nc*CBH I and rh*Pf*CBH I, respectively; *lanes 2* and *4*, corresponding rhCBH I treated by endo-H; **b** *lanes 1* and *2*, rh*Tr*CBH II and endo-H treated rh*Tr*CBH II, respectively

CBH I and CBH II (Cel7A and Cel6A) are the major exo-acting components of the *T. reesei* cellulolytic secretome, representing 50 and 20% of the total amount of the protein respectively (w/w) [31]. However, type I cellobiohydrolases from various fungal sources have so far proved to be difficult to express in heterologous hosts, such as *S. cerevisiae* and *Y. lipolytica*, probably due to the improper folding and/or unnatural post-translation patterns [27, 38, 39]. Taking this into account, we attempted to express in *Y. lipolytica* three different CBH I from *T. reesei* (*Tr*CBH I), *Penicillium funiculosum* (*Pf*CBH I) and *Neurospora crassa* (*Nc*CBH I). It is noteworthy that the latter two have been shown to possess quite potent cellulose-degrading activities [40–42]. Accordingly, *Tr*CBH I*, Pf*CBH I and *Nc*CBH I were produced in *Y. lipolytica* as His-tagged proteins (rh*Tr*CBH I, rh*Pf*CBH I and rh*Nc*CBH I) using the TEF promoter and the *Y. lipolytica* lipase 2 pre-pro region. Subsequent SDS-PAGE and anti-His Western blot analyses of the culture supernatants indicated that rh*Tr*CBH I could only be detected as a faint band, consistent with previous results [27] (data not shown). Contrastingly, the successful expression of rh*Nc*CBH I and rh*Pf*CBH I was confirmed by the clear presence of new protein species. In the case of rh*Nc*CBH I a Mw of approximately 75 kDa was determined, while expression of rh*Pf*CBH I produced a smear in the Mw range 70–200 kDa. Since the theoretical Mw of *Pf*CBH I and *Nc*CBH I is 52 kDa each, it was possible to deduce that rh*Pf*CBH I and rh*Nc*CBH I are glycosylated (Fig. 3a). Therefore, along with rh*Tr*CBH I, rh*Pf*CBH I and rh*Nc*CBH I were submitted to EndoH-mediated deglycosylation. This yielded protein products with Mw of approximately 75 kDa, suggesting that the enzymes might bear other post-translational modifications other than N-glycosylation (Fig. 3a). Further analysis using an activity assay revealed that the Avicelase activity in the culture supernatant of the transformant yl*Nc*CBH I (0.01 U/mL) was 10 times higher than that in the supernatant of yl*Pf*CBH I. Avicelase activity lower than the minimum detectable limit (0. 001 U/mL) was found in the case of yl*Tr*CBH I. Based upon this simple screening approach, *Nc*CBH I was retained as the CBH I component for future work.

Regarding the requirement for CBH II, the *T. reesei* enzyme was chosen, because previous work has already shown that this enzyme can be satisfactorily produced in *Y. lipolytica* [26, 27]. In the present work, Western blot analysis indicated that rhTrCBH II bears post-translational modifications, since its apparent Mw is 75 kDa, which is higher than the theoretical value of 48 kDa. Deglycosylation of rhTrCBH II using EndoH revealed that this protein contains N-linked glycosylation, yet, its Mw remains slightly superior to 48 kDa after this enzymatic treatment (Fig. 3b). This could possibly be related to the presence of linker regions, rich in serine and threonine which are often highly O-glycosylated, connecting *T. reesei* CBH II catalytic domain and carbohydrate-binding module. Finally, it is noteworthy that the expression of the different rCBHs without the His-tag yielded similar activities on Avicel and PASC when culture supernatants were tested (i.e,. comparison of unpurified protein preparations).

### Characterization of the recombinant cellobiohydrolases expressed in *Y. lipolytica*

**Fig. 4:**
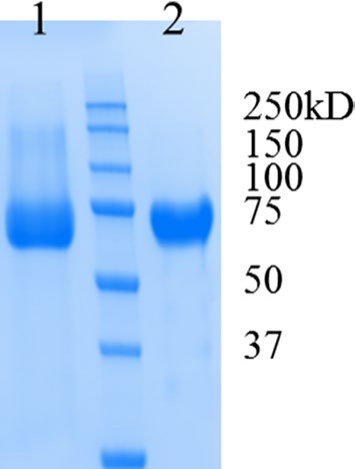
SDS-PAGE analysis of the purified rh*Nc*CBH I (*lane 1*) and rh*Tr*CBH II (*lane 2*) produced by *Y. lipolytica* JMY1212

In order to investigate the cellulose-degrading abilities of rh*Nc*CBH I and rh*Tr*CBH II, the recombinant CBHs were purified and characterized. Purification was achieved in a single step using IMAC with both proteins being obtained in good yields (>60% of the expressed protein) (Fig. 4). Afterward, each enzyme was tested for its ability to hydrolyze various substrates (Table 2). The specific activities of rh*Nc*CBH I on Avicel and PASC were two and four times that of the reported values for native *Tr*CBH I, respectively [43], that is to say two times the amount of the only cellobiohydrolase of family I expressed in *Y. lipolytica* [27]. In contrast, the comparison of native and rh*Tr*CBH II revealed similar specific activities on these substrates, consistent with the previously reported values [26, 43]. The high Avicelase activity of rh*Nc*CBH I is noteworthy, because Avicel is known to be crystalline and quite recalcitrant to enzyme hydrolysis. Likewise, the specific activity of rh*Nc*CBH I on amorphous PASC is four times that of native *Tr*CBH I and CBH II (Table 2). A recent study has illustrated that *Tr*CBH I remains poorly active on cellulosic substrates until amorphous regions in the cellulose substrate are removed by *Tr*CBH II [44]. Nevertheless, in the case of a cocktail containing *Nc*CBH I, this limitation might be somehow attenuated by the superior activity of this CBH I on amorphous cellulose. Because it is known that the exo–exo synergy between type I and type II CBHs is crucial for efficient cellulose degradation, coexpression of *Nc*CBH I and *Tr*CBH II is expected to satisfy this criterion [45].

**Table 2:** Comparison of the hydrolytic activities of purified His-tagged *Nc*CBH I and *Tr*CBH II expressed in *Y. lipolytica* with native *T. reesei* CBHs on various cellulosic substrates

	Specific activity (μmol/min/mg)	
	Avicel	PASC
*Nc*CBH I	0.11	2.5
Native *Tr*CBH I [43]	0.065	0.6
*Tr*CBH II	0.056	0.6
Native *Tr*CBH II [43]	0.065	0.6

The mean value of three independent experiments is shown, and the standard deviation is less than 10%

Finally, based on the measurement of total PASCase activities in the culture supernatant and specific activities of the rhCBH enzymes, it was possible to determine that the amount of rh*Nc*CBH I and rh*Tr*CBH II secreted by *Y. lipolytica* in YTD medium was approximately 24 and 75 mg/L, respectively. Regarding rh*Tr*CBH II, these results are consistent with those of a previous study in which it was expressed without a His-tag in *Y. lipolytica* [26, 27].

### Enhancement of cellobiohydrolase production in *Y. lipolytica* using hybrid promoter

**Fig. 5:**
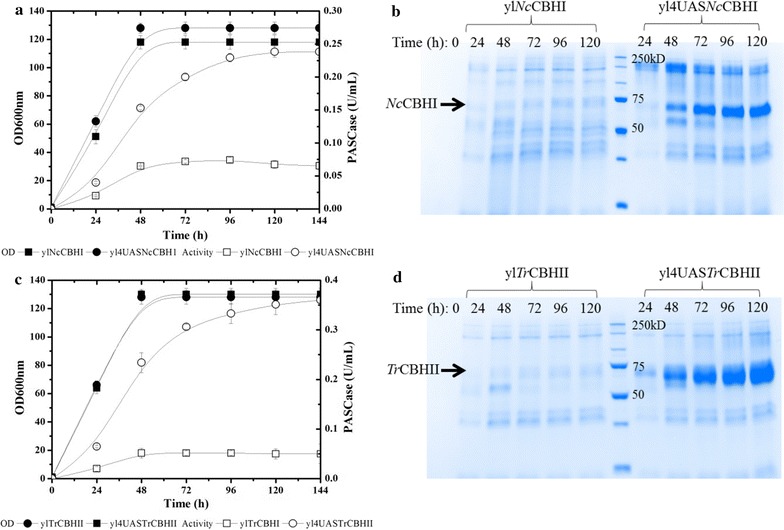
Production of rh*Nc*CBH I and rh*Tr*CBH II under the control of *TEF* or *4UASTEF* promoter in *Y. lipolytica* **a** and **c** enzyme production on YTD vs. time; **b** and **d** SDS-PAGE analysis of the culture supernatant of *Y. lipolytica* transformants

Several studies have shown that it is important to ensure that a sufficient amount of cellobiohydrolases are present in LCB-active cocktails in order to promote synergy with EGs and BGLs [33–35]. Therefore, to enhance the expression of r*Tr*CBH II and r*Nc*CBH I, their encoding sequences were placed under the control of the hybrid promoter (HTEF), which is composed of four tandem copies of upstream activation sequences (UAS) and the core promoter element of TEF [46]. Monitoring PASCase activity and using SDS-PAGE revealed that while the TEF-controlled production of r*Nc*CBH I was highly cell growth-dependent, reaching its highest level at the beginning of stationary phase (Fig. 5a, b), HTEF-controlled production was much higher and continued to increase over the 5-day growth period. Importantly, when using the HTEF promoter, the final yield of rh*Nc*CBH I (95 mg/L) was 4 times higher than that obtained using TEF and was much higher than previously reported yields [27]. Similarly, when rh*Tr*CBH II was produced under the control of HTEF, the yield of this protein was eightfold higher (600 mg/L) than that obtained when using the TEF promoter (Fig. 5c). This significant increase was also evidenced upon SDS-PAGE (Fig. 5d). In this regard, although previous studies have already demonstrated the benefits of using the hybrid promoter to enhance protein production in *Y. lipolytica*, studies so far have only been focused on intracellular proteins [46]. Here we supply two examples of enhanced extracellular production and further reveal that, based on the findings of Blazeck et al. [46], the production level of *Tr*CBH II surpasses expectations, with the use of four tandem copies of UAS yielding a fourfold increase in protein production.

### Construction of recombinant *Y. lipolytica* strains expressing different combinations and ratios of cellulases

**Fig. 6:**
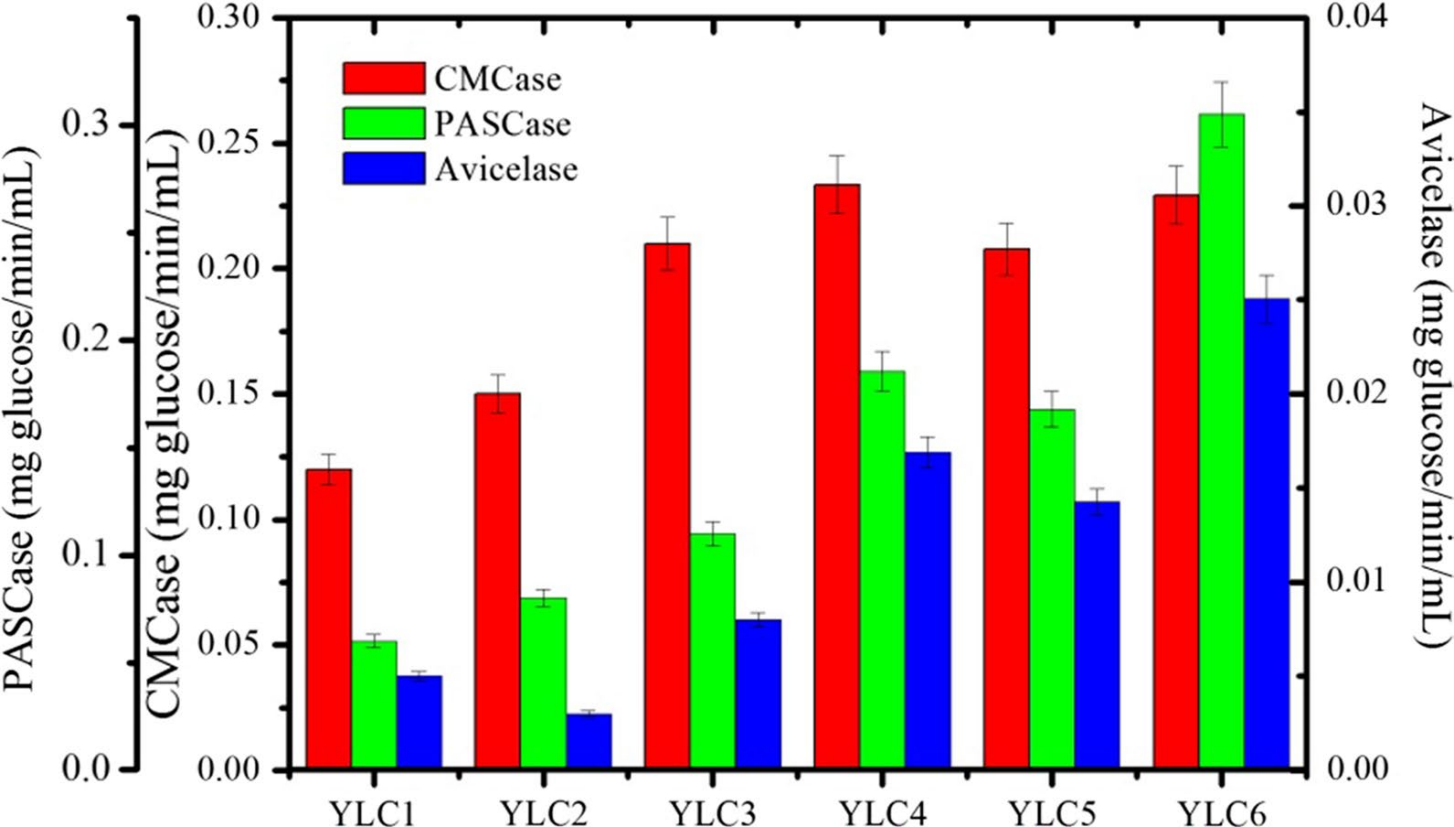
Comparison of the hydrolytic activities of the total secreted cellulases produced by different cellulolytic *Y. lipolytica* strains cultivated in Y_1_T_2_D_5_ media after 120 h on various cellulosic substrates

To confer optimal cellulose-degrading ability to *Y. lipolytica*, several strains were constructed, using the previously developed BGL-producing strain as a platform [28]. Moreover, to identify the best configuration, strains were built using different enzyme combinations and protein production ratios (Table 3). The success of introduction of different cellulase-encoding genes into *Y. lipolytica* was verified by PCR (Additional file 1: Figure S2). The positive clones were chosen based on the results of enzymatic activity assays (data not shown). Subsequently, these selected strains were cultivated in YTD medium and the hydrolytic activities of the total secretory secreted proteins were analyzed on cellulosic substrates CMC, PASC and Avicel (Fig. 6).

**Table 3:** Microbial strains used in the present study

Strains	Relevant genotype	Source of reference
*T. reesei* QM9414	Wild type	DSMZ
*E. coli* DH5	Φ80dlacZΔm15, *recA1*, *endA1*, *gyrA96*, *thi*-*1*, *hsdR17* (rk^−^, mk^+^), *supE44*, *relA1*, *deoR*, Δ(*lacZY*A-argF) U169	Invitrogen
*Y. lipolytica* JMY1212 (Zeta)	*MATA*, *ura3*-*302*, *leu2*-*270*-*LEU2*-*zeta*, *xpr2*-*322 ∆lip2*, *∆lip7*, *∆lip8*	[30]
*Y. lipolytica* ∆*pox*B12	*MATA, xpr2*-*322, pox1*-*6∆, pTEF*-*BGL1, pTEF*-*BGL2*	[28]
ylTrEGI	Zeta*, pTEF*-*EG I*-*His6*	This investigation
ylTrEGII	Zeta*, pTEF*-*EG II*-*His6*	This investigation
yl*Nc*CBHI	Zeta*, pTEF*-*CBH I*-*His6*	This investigation
yl4UAS*Nc*CBHI	Zeta*, pHTEF*-*CBH I*-*His6*	This investigation
yl*Tr*CBHII	Zeta*, pTEF*-*CBH II*-*His6*	This investigation
yl4UAS*Tr*CBHII	Zeta*, pHTEF*-*CBH II*-*His6*	This investigation
YLC1	∆*pox*B12, *pTEF*-*EG I, pTEF*-*CBH I, pTEF*-*CBH II*	This investigation
YLC2	∆*pox*B12, *pTEF*-*EG II, pTEF*-*CBH I, pTEF*-*CBH II*	This investigation
YLC3	∆poxB12, *pTEF*-*EG I*, *pTEF*-*EG II*, *pTEF*-*CBH I*, *pTEF*-*CBH II*	This investigation
YLC4	∆poxB12, *pTEF*-*EG I*, *pTEF*-*EG II*, *pTEF*-*CBH I*, *pHTEF*-*CBH II*	This investigation
YLC5	∆*pox*B12, *pTEF*-*EG I, pTEF*-*EG II, pHTEF*-*CBH I*, *pTEF*-*CBH II*	This investigation
YLC6	∆*pox*B12, *pTEF*-*EG I, pTEF*-*EG II, pHTEF*-*CBH I, pHTEF*-*CBH II*	This investigation

The strain YLC3, expressing two r*Tr*EGs, displayed higher CMCase activity than YLC1 and YLC2, which express either r*Tr*EG I or r*Tr*EG II, alone (Fig. 6). Moreover, quite predictably, strain YLC1 expressing only r*Tr*EG I and two CBHs, was more active on Avicel than YLC2, which is a homolog that expresses r*Tr*EG II instead of r*Tr*EG I. Consistently, YLC2 displayed better activity on PASC. When both r*Tr*EG I and II are present (strains YLC3-6), the hydrolyses of Avicel and PASC were enhanced, demonstrating that the presence of both EGs is necessary to achieve optimal cellulolytic activity. In contrast, the ability of strain YLC3 to hydrolyze CMC was similar to that of strains YLC4-6, implying that the basic combination of the six enzymes is sufficient to achieve optimal results with this substrate. On the other hand, increasing the expression level of either r*Tr*CBH II (YLC4) or r*Nc*CBH I (YLC5) using the HTEF promoter resulted in improved Avicel and PASC hydrolyses. Further increases of Avicel and PASC hydrolyses were achieved by enhancing the expression levels of both *Nc*CBH I and *Tr*CBH II (YLC6). The presence and the abundance of each expressed enzyme in YLC6 were confirmed by proteomics analysis (Additional file 2), which revealed that the ratio between each cellulase component in the secretome of YLC6 is consistent with the ratio of respective protein individually produced by recombinant *Y. lipolytica* (Plateforme Protéomique de la Génopole Toulouse Midi-Pyrénées, IPBS, France). Overall, the performance of YLC6 was rather encouraging. However, accounting for the fact that Avicel and PASC are model substrates, our conclusions cannot be further extrapolated to predict the aptitude of YLC6 for use on industrial cellulose pulps, because these are generally of a much more complex nature [47].

### CIMV-cellulose and Avicel fermentation with recombinant *Y. lipolytica* strains

In order to explore the potentiality of the *Y. lipolytica* strains developed in this study for use as CBP microorganisms, these were grown in defined minimum medium containing either industrial cellulose pulp (CIMV, cellulose content ≈91%) or Avicel as the carbon source. Gratifyingly, all of the strains consumed CIMV-cellulose, although Avicel was less amenable to hydrolysis. The strain YLC3 expressing all the essential cellulase components used 40% of the CIMV-cellulose provided (27.5 g/L, initial concentration, i.e,. 25 g/L cellulose) and produced 0.38 g DW cells/(g substrate). These values increased to approximately 50% and 0.40 g DW cells/(g substrate) in the case of YLC4 and 5, and to 59% and 0.41 g DW cells/(g substrate) in the case of YLC6. Overall, these results demonstrate that cellulolytic capacity had been successfully conferred to *Y. lipolytica* and support findings that CIMV-cellulose is mainly amorphous and highly amenable to enzyme-mediated cellulolysis. However, our results also highlight the highly recalcitrant nature of Avicel, since the final consumption of this substrate by strains YLC4-6 (26–30%) was not much higher than that achieved by YLC3 (22%) or that procured by a coculturing approach (23%) [27], where each cellulase component was produced separately (Table 4). Significantly, even prolonged growth periods did not enhance Avicel degradation, indicating that the cellulase combinations used in this study are inadequate for the hydrolysis of crystalline cellulose. By contrast, as an attractive microorganism for CBP, *C. thermocellum* displays remarkable capacity toward the hydrolysis of crystalline cellulose for which the highest Avicel cellulose consumption rate of 0.5 g/L/h was reported recently [16], which is 7 times higher than the one obtained in YLC6. In addition to substrate crystallinity, it is clear that the performance of the strains was also hampered by the amounts of enzyme activity available in the culture medium, which were lower than those achieved when using richer YTD medium (data not shown). Beyond the penalizing effect of the minimal medium on protein expression, it is important to note that the enzymes were also operating in suboptimal pH and temperature conditions. This is because they were deployed in a simultaneous hydrolysis and fermentation reaction that was necessarily conducted at the optimal conditions for yeast growth. Therefore, in future work it will be necessary to address this issue, perhaps through the use of enzymes that are better adapted to low temperature that characterize the optimal growth conditions of *Y. lipolytica*.

**Table 4:** Comparison of cellulose utilizations and biomass yields of cellulolytic *Y. lipolytica* grown for 120 h in aerobic cultivation on CIMV-cellulose (27.5 g/L) and Avicel (25 g/L)

Strains	CIMV-cellulose consumed %	Biomass yield (g-DCW/g-CIMV consumed)	Avicel consumed %	Biomass yield (g-DCW/g-Avicel consumed)
YLC1	30.5	0.36	19.6	0.29
YLC2	36.8	0.36	17.2	0.26
YLC3	40.2	0.37	22.0	0.30
YLC4	50.4	0.40	27.1	0.31
YLC5	52.0	0.40	26.3	0.30
YLC6	58.6	0.41	30.2	0.32

The mean value of three independent experiments is shown, and the standard deviation is less than 10%

## Conclusions

In this article, we have provided a clear demonstration of how cellulolytic activity can be conferred to *Y. lipolytica*, thus opening the way toward a consolidated bioprocess. Using the panoply of available tools, including strong artificial promoters and selected cellulase components, it has been possible to show that *Y. lipolytica* can achieve high protein expression levels and that even when the enzymes function in suboptimal temperature conditions, their production and activity are sufficient to allow cellulose hydrolysis. In particular, it is noteworthy that the heterologous expression of CBH I from *N. crassa* was particularly successful and that this enzyme is more active on Avicel than its *T. reesei* counterpart. This is significant because CBH I is essential for the degradation of recalcitrant LCB.

Options for future improvements to the strains described herein include the introduction of lytic polysaccharide monooxygenases, the adaptation of the operating conditions of the enzymes to suit the growth conditions of *Y. lipolytica* or, alternatively, the enhancement of the thermal resistance of the host yeast strain. Overall, these encouraging findings confirm that the creation of an efficient, engineered cellulolytic *Y. lipolytica* strain is achievable. Moreover, the good performance of our prototype strain on a sample of industrial cellulose substrate reveals that such a strain could be a useful starting point for the development of an advanced generation biorefinery process for the production of bioenergy and valuable chemicals. In addition to the future steps described above, a further action will be to demonstrate that a cellulolytic *Y. lipolytica* strain can produce lipids when growing on cellulose as the sole carbon source.

## Methods

### Strains and media

The genotypes of the microbial strains used in the present study are summarized in Table 3. *E. coli* DH5 were purchased from Invitrogen (Paisley, UK) and used for plasmid construction. The *Y. lipolytica* strains were routinely cultivated in a medium composed of 10 g/L yeast extract, 20 g/L Bacto peptone, and 20 g/L glucose (YPD). Transformants were selected on solid YNB medium (1.7 g/L YNB, 10 g/L glucose or cellobiose, 5 g/L ammonium chloride, with (for Ura^+^) or without (for Leu^+^) 2 g/L casamino acids, and 50 mM sodium–potassium phosphate buffer, pH 6.8), supplemented with uracil (440 mg/L) or leucine (440 mg/L) depending on the auxotrophic requirements. Solid media contained 1.5% agar. The detection of endoglucanase activity in solid YNBcasa medium was achieved by incorporating 2 g/L Azo-CM-Cellulose (Megazyme). For cellulase characterization, enzymes were produced in YTD medium (10 g/L, 20 g/L tryptone, 50 g/L glucose, and 100 mM phosphate buffer, pH 6.8). To evaluate the growth of the engineered cellulolytic *Y. lipolytica* on cellulose, transformants were aerobically cultivated in defined medium containing vitamins, trace elements [48], and salts, including 3.5 g/L (NH_4_)_2_SO_4_, 3.0 g/L K_2_HPO_4_, 3.0 g/L NaH_2_PO_4_, and 1.0 g/L MgSO_4_·7H_2_O with 27 g/L CIMV-cellulose (cellulose content ≈91%, provided by CIMV S.A.) or 25 g/L Avicel PH-101 (Sigma).

### Plasmid constructions

The plasmids constructed in the present study are summarized in Table 5, and all primers are listed in Table 6. In brief, the total RNA from 5-day-cultured *T. reesei* QM9414 was isolated using RNeasy Plus Mini Kit (QIAGEN), and reverse transcription was performed using iScript™ cDNA Synthesis Kit (BIO-RAD) according to the manufacturer’s instructions. For the expression of wild-type proteins, EG I (GenBank accession code: XM_006965612.1), EG II (GenBank accession code: XM_006965612.1), and CBH II (GenBank accession code: XM_006962518.1) were amplified from the cDNA of *T. reesei* by PCR using F (1-3) as forward primers and R (1-3) as reverse primers, respectively. A 15-base pair homologous sequence of the target plasmid was introduced into the end of each gene during PCR amplification. Afterward, the genes encoding EG I, EG II, and CBH II were fused with the PCR fragment (primers JMP1F/JMP1R) of secretion vector JMP62UraTEF or JMP62LeuTEF under the control of TEF promoter and the pre-pro sequence of lipase 2 (33N-terminal amino acids of Lipase 2, Genbank accession number: Q9P8F7) of *Y. lipolytica* by In-Fusion Cloning Kits (Clontech). For the expression of His-tagged proteins, EG I, EG II, and CBH II were cloned by PCR with F4 as forward primer and R (4-6) as reverse primers using the expression vectors constructed in last step as template, and fused with PCR fragment (primers JMP2F/JMP2R) of the vector JMP62UraTB1his [28].

**Table 5:** Plasmids used or created in the present study

Plasmids	Description	Source of reference
JMP62UraTEF	*URA3*, *pTEF*	[25]
JMP62LeuTEF	*LEU2*, *pTEF*	[25]
JMP62UraTB1his	*URA3*, *pTEF*-*BGL1*-*His6*	[28]
PUB4-CRE	*hph, hp4d*-*CRE*	[49]
JMP62UraHTEF	*URA3*, *pHTEF*	This investigation
JMP62LeuHTEF	*LEU2*, *pHTEF*	This investigation
JMP62UraTrEG1	*URA3*, *pTEF*-*TrEG I*	This investigation
JMP62LeuTrEG2	*LEU2*, *pTEF*-*TrEG II*	This investigation
JMP62UraNcCBH1	*URA3*, *pTEF*-*NcCBH I*	This investigation
JMP62LeuTrCBH2	*LEU2*, *pTEF*-*TrCBH II*	This investigation
JMP62UraHNcCBH1	*URA3*, *pHTEF*-*NcCBH I*	This investigation
JMP62LeuHTrCBH2	*LEU2*, *pHTEF*-*TrCBH II*	This investigation

**Table 6:** Sequences of the oligonucleotide primers used in this study

Primer names	Sequence (5′-3′), 15-bp homologous sequence for infusion is underlined
F1	GTTCTCCAGAAGCGACAGCAACCGGGTACCAGCAC
R1	CACAGACACCCTAGGCTAAAGGCATTGCGAGTAGTAGTCGT
F2	GTTCTCCAGAAGCGAGCACAGCAGACTGTCTGGGGCC
R2	CACAGACACCCTAGGCTACTTTCTTGCGAGACACGAGCTGAC
F3	GTTCTCCAGAAGCGAGCCCAGGCTTGCTCAAGCGTC
R3	CACAGACACCCTAGGTTACAGGAACGATGGGTTTGCGT
JMP1F	CCTAGGGTGTCTGTGGTATCTAAGCTATT
JMP1R	TCGCTTCTGGAGAACTGCGG
F4	ACACCCGAAGGATCCCACAATGAAGCTTTCCACCATCC
R4	ATGGTGATGATGGTGAAGGCATTGCGAGTAGTAGTCGT
R5	ATGGTGATGATGGTGCTTTCTTGCGAGACACGAGCTGAC
R6	ATGGTGATGATGGTGCAGGAACGATGGGTTTGCGT
JMP2F	CACCATCATCACCATCATTAAAACT
JMP2R	GGATCCTTCGGGTGTGAGTTG
HTF	ATCCCTAGAATCGATGCCGCCGCAAGGAATGG
HTR	GCCAACCCGGTCTCTGCACTTTTGCCCGTGATCAGTG
JMP3F	AGAGACCGGGTTGGCGG
JMP3R	ATCGATTCTAGGGATAACAGGGTAATT

Considering the challenge to express CBH I proteins in yeast, the gene coding the sequences of CBH I from *T. reesei*, *P. funiculosum*, and *N. crassa* were codon-optimized based on the codon bias of *Y. lipolytica*, and were synthesized by GenScript (USA) and cloned into the plasmid JMP62UraTEF, JMP62LeuTEF, or JMP62UraTB1his under the control of TEF promoter. These codon-optimized nucleotide sequences can be found in Additional file 3.

In addition, an enhancer comprising four tandem copies of upstream activation sequences (4UASs) was cloned by PCR with primers HTF and HTR using vector PUB4-CRE as template [49]. The fusion of this DNA fragment with the PCR product (primers JMP3F/JMP3R) of vector JMP62UraTEF and JMP62LeuTEF yielded vectors JMP62UraHTEF and JMP62LeuHTEF, respectively. To enhance the expression level of CBH proteins, the plasmids, JMP62UraCBHI and JMP62LeuCBHII, were digested using *Bam*HI/*Avr*II, and the *Nc*CBH I and *Tr*CBH II genes were, respectively, recovered and then inserted into the corresponding sites of the plasmids, JMP62UraHTEF and JMP62LeuHTEF.

After construction, all expression vectors were verified by DNA sequencing (GATC Biotech, Konstanz, Germany). For *Y. lipolytica* transformation, vectors were digested using *Not*I, thus generating a linear DNA with Zeta sequences at both extremities. Then, the gel-purified expression cassettes were introduced into the Zeta docking platform of *Y. lipolytica* JMY1212 Zeta for the expression of single cellulase, or randomly into the genome of ∆*pox*B12 strain, for coexpression of multiple cellulases, using the lithium acetate method [50]. For the latter case, the *LoxP*-Cre recombination system was used for marker rescue and to ensure the multistep insertion of the target genes [49]. The successful multiple integration of the heterologous genes into the genome of *Y. lipolytica* was verified by PCR using gene-specific primers (Additional file 1: Table S1). In addition, transformants expressing multiple enzymes were tested for growth on cellobiose, and for degradation of Azo-CMC. Clones displaying both activities were retained for further analysis. Table 3 summarizes the expressed cellulase genes and their corresponding *Y. lipolytica* transformants.

### Enzyme production and activity assay

Recombinant protein production by *Y. lipolytica* was carried in YTD medium in shake flask at 28 °C and 120 rpm for 5 days. PASC was prepared from Avicel PH-101 as previously described [51]. The activities of EGs were measured on CMC (Megazyme), PASC, β-1, 3-glucan from *Euglena gracilis* (≥99%, Sigma), β-1, 4-glucan from barley (≥95%, Sigma), Avicel PH-101 (Sigma), and cellotriose (Megazyme) using previously described method with slight modifications [52]. In brief, the reaction mixture contained 1% (w/v) insoluble cellulosic substrate, or 5 mM cellotriose, 50 mM citrate buffer (pH 4.8), and proper volume of diluted enzyme solution. The reaction was conducted at 50 °C for 30 min, and then reducing sugars were quantified using the dinitrosalicylic acid (DNS) reagent. A similar method was used to evaluate CBH activity using PASC and Avicel as substrates. One unit of activity (U) was defined as the amount of enzyme required to release 1 μmol of reducing sugars per min. All protein concentrations were measured using the Bradford method and bovine serum albumin as a standard [53]. All enzymatic activity measurements were performed in triplicate unless otherwise stated.

### SDS-PAGE and Western blot analysis

SDS-PAGE was conducted using Mini-PROTEAN TGX Stain-Free precast gels (Biorad) according to the manufacturer’s instructions. 15 μL of culture supernatant or enzyme solution was loaded into each well. Western blotting of proteins was performed as described previously [54]. Crude supernatant of *Y. lipolytica* JMY1212 expressing EGs and CBHs fused with the His6 tag were concentrated 10-fold by ultrafiltration with an Omega™ membrane disk filter at 10 kDa cut off (Pall, France). Blots were sequentially treated with mouse non position-specific His-Tag antibody 1:2500 (THE™ from Genscript, Piscataway, NJ, USA) and the alkaline phosphatase-conjugated goat anti-mouse IgG (1:10000). For the detection, the PVDF membrane was incubated with a mixture of nitro blue tetrazolium chloride and 5-Bromo-4-chloro-3-indolyl phosphate (NBT/BCIP) (Sigma).

### Purification of recombinant cellulases and deglycosylation

*Yarrowia lipolytica* JMY1212 expressing His6-tagged EG I, EG II, CBH I, and CBH II, respectively, was grown in 200 mL YTD medium at 120 rpm, 28 °C for 48 h. After centrifugation (8000×*g* for 5 min at 4 °C), the supernatant was concentrated 10-fold by ultrafiltration using an Omega™ membrane disk filter at 10 kDa cutoff (Pall, France), and applied to 2 mL of TALON Metal Affinity Resin (Clontech, Takara-Bio, Kyoto, Japan). Subsequently, protein was eluted using imidazole buffer according to the manufacturer’s instructions. Deglycosylation was carried out by treating the purified proteins with endoglycosidase H (New England Biolabs, Beverly, MA, USA) to remove N-linked carbohydrates at 37 °C for 1 h. Protein samples were analyzed by SDS-PAGE and visualized with colloidal coomassie blue staining.

### Growth of yeast expressing multiple cellulases on CIMV and Avicel cellulose

Yeast growth on CIMV-cellulose and Avicel was performed in 50 mL of the defined medium containing 27.5 g/L CIMV or 25 g/L Avicel cellulose stored in 250-mL Erlenmeyer flasks. Yeasts were precultivated in defined medium until middle exponential phase, and the cells were collected by centrifugation. After washing with deionized water, the cells were used to inoculate the defined medium to yield an initial biomass concentration of 1.0 g-DCW/L. The cultivations were conducted at 28 °C, and samples were taken at the end of 5 days to determine concentrations of biomass and residual cellulose.

### Analysis of residual cellulose and determination of dry cell weight

The quantification of cellulose residues and dry cell matter was conducted as previously described with slight modifications [27]. In brief, the cell pellets mixed with cellulose residues from the above cultures were harvested by centrifugation at 8000×*g* for 10 min at 4 °C. After 2-time wash with distilled water, the collected cellulose–cell pellet was freeze-dried and weighed. The amount of cellulose that remained was calculated from the total glucose released from enzymatic hydrolysis of the cellulose residues using Cellic^®^ CTec2, and verified by diluted acid-based hydrolysis of the residues with 2.5% sulfuric acid at 121 °C for 1 h. Dry cell weight was deduced by subtracting the amount of cellulose from the weight of cellulose–cell pellet. Glucose was measured by HPLC as described before [28]. The biomass yield was calculated as the ratio of the amount of biomass obtained divided by the amount of carbon source consumed.

## Additional files

**Additional file 1**: **Figure S1.** Screening of *Y. lipolytica* expressing EGs on indication plate containing YNBcasa medium supplemented with 0.2% w/vAzo-CM-Cellulose. **Figure S2.** PCR verification of *Y. lipolytica* transformants expressing multiple cellulases. **Figure S3.** Nucleotide sequences of constructs. **Table S1.** The sequences of the oligonucleotide primers used for PCR verification of yl-transformants.

**Additional file 2.** Proteomics analysis of the de-glycosylated secretome of recombinant strain YLC6.

**Additional file 3.** Nucleotide sequences of constructs.

## Abbreviations

BGL: β-glucosidase
CBH: cellobiohydrolase
CBP: consolidated bioprocessing
CMC: carboxymethyl cellulose
DCW: dry cell weight
EG: endoglucanase
Endo H: endoglycosidase
FDA: Food and Drug Administration
GRAS: generally recognized as safe
rh: His6-tagged recombinant proteins
LCB: lignocellulosic biomass
MW: molecular weight
*Nc*: *Neurospora crassa*
PAGE: polyacrylamide gel electrophoresis
PASC: phosphoric acid-swollen cellulose
*Pf*: *Penicillium funiculosum*
*r*: recombinant proteins
*Tr*: *Trichoderma reesei*
*yl*: *Y. lipolytica*
