# Supplementary material for: Conferring cellulose-degrading ability to Yarrowia lipolytica to facilitate a consolidated bioprocessing approach
Source: Biotechnol Biofuels. 2017 May 19;10:132. doi: 10.1186/s13068-017-0819-8 (PMC5438512; doi:10.1186/s13068-017-0819-8)
Supplement: Supplementary file 1 — Additional file 1: Figure S1. Screening of Y. lipolytica expressing EGs on indication plate containing YNBcasa medium supplemented with 0.2% w/vAzo-CM-Cellulose. Figure S2. PCR verification of Y. lipolytica transformants expressing multiple cellulases. Figure S3. Nucleotide sequences of constructs. Table S1. The sequences of the oligonucleotide primers used for PCR verification of yl-transformants. [file 13068_2017_819_MOESM1_ESM.pdf]

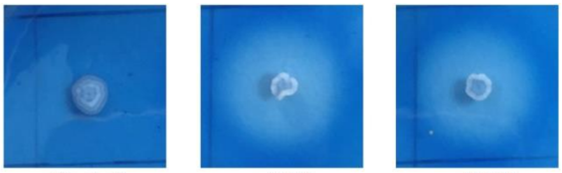

Figure S1 Screening of *Y. lipolytica* expressing EGs on indication plate containing YNBcasa medium supplemented with 0.2% w/vAzo-CM-Cellulose.

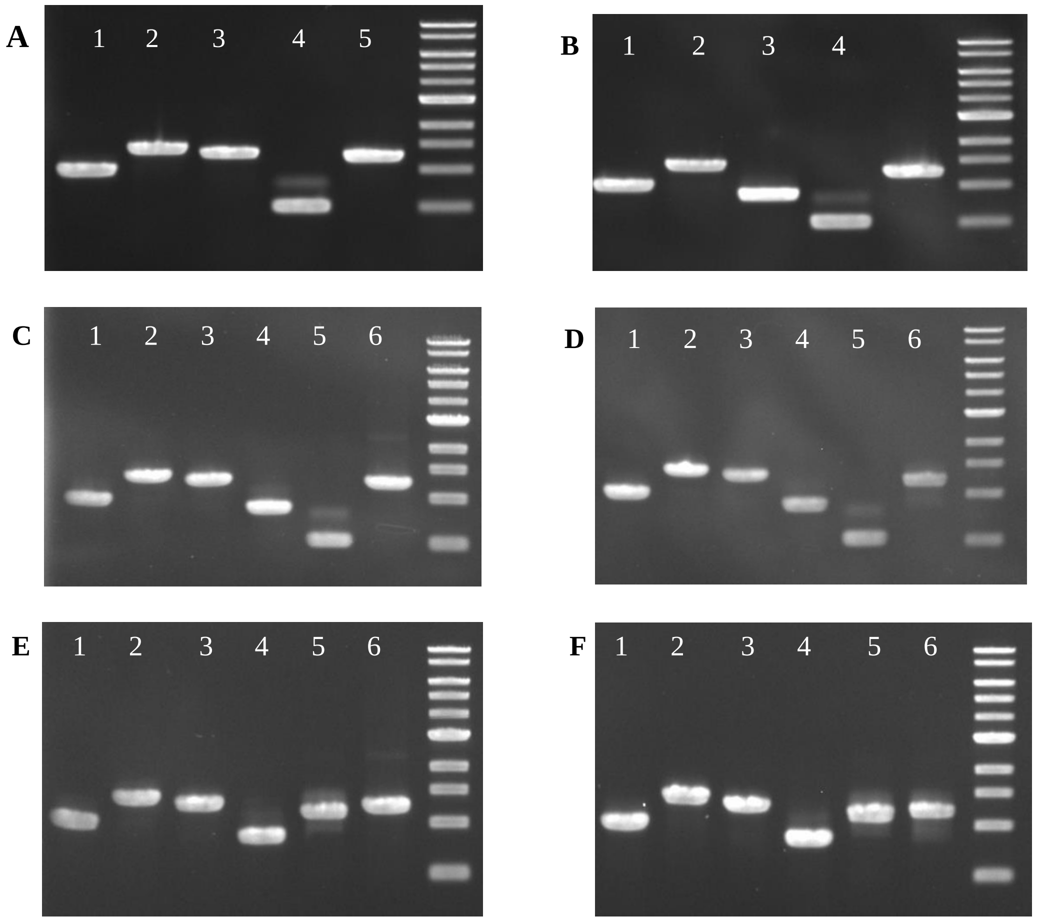

Figure S2 PCR verification of *Y. lipolytica* transformants expressing multiple cellulases (A) YLC1, Lane 1, 2, 3, 4, 5: *BGL1*, *BGL2*, *TrEGI*, *NcCBHI*, *TrCBHII*; (B) YLC2, Lane 1, 2, 3, 4, 5: *BGL1*, *BGL2*, *TrEGII*, *NcCBHI*, *TrCBHII*; (C) YLC3, Lane 1, 2, 3, 4, 5, 6: *BGL1*, *BGL2*, *TrEGI*, *TrEGII*, *NcCBHI*, *TrCBHII*; (D) YLC4, Lane 1, 2, 3, 4, 5, 6: *BGL1*, *BGL2*, *TrEGI*, *TrEGII*, *NcCBHI*, *4UASTrCBHII*; (E) YLC5, Lane 1, 2, 3, 4, 5, 6: *BGL1*, *BGL2*, *TrEGI*, *TrEGII,4UASNcCBHI*, *TrCBHII*; (F) YLC6, Lane 1, 2, 3, 4, 5, 6: *BGL1*, *BGL2*, *TrEGI*, *TrEGII*, *4UASNcCBHI*, *4UASTrCBHII*.

Table S1 The sequences of the oligonucleotide primers used for PCR verification of yl-transformants

Primer names	Sequence (5’-3’)
VBGL1A	TTGACCCAGTAGCGGACCCAA
VBGL1B	GCCGACATTAGCCCTAACAGCAT
VBGL2A	GGTTGGCGGCGCATTTGT
VBGL2B	TGTCGTCCACTCGGCTTTCATC
VEGIA	CACTTGCCGTTAAGGGCGTAGGGT
VEGIB	CTGGCTGTTGTCGTTCCAAATGCTG
VEGIIA	CTTGCCGTTAAGGGCGTAGGG
VEGIIB	CCATCGAGCATAATTGTGGATGTCG
VCBHIA	TTTGCTTTGTGGTTGGGACTTTAGC
VCBHIB	GGTTCGAGATCCGATGTTAGTGGAGTA
VCBHIIA	TTTGCTTTGTGGTTGGGACTTTAGCC
VCBHIIB	AGGCGTTGGACCAGCCGTGA
V4UASCBHIA	CGCCGCAAGGAATGGTGCA
V4UASCBHIB	GGTTCGAGATCCGATGTTAGTGGAGTA
V4UASCBHIIA	CGCCGCAAGGAATGGTGCA
V4UASCBHIIB	CCGATCCGACTGGAGGTACTCTGGTAG
