# Supplementary material for: Conferring cellulose-degrading ability to Yarrowia lipolytica to facilitate a consolidated bioprocessing approach
Source: Biotechnol Biofuels. 2017 May 19;10:132. doi: 10.1186/s13068-017-0819-8 (PMC5438512; doi:10.1186/s13068-017-0819-8)
Supplement: Supplementary file 3 — Additional file 3. Nucleotide sequences of constructs. [file 13068_2017_819_MOESM3_ESM.pdf]

Nucleotide sequences of constructs

Note that these nucleotide sequences were codon-optimized based on the codon bias of *Y. lipolytica* and are started with prepro sequence of Lip2 of *Y. lipolytica* (underlined)

>*Tr*CBH1

ATGAAGCTTTCCACCATCCTTTTCACAGCCTGCGCTACCCTGGCTGCCGCCCTCCCTTC
CCCCATCACTCCTTCTGAGGCCGCAGTTCTCCAGAAGCGACAGTCGGCCTGCACTCTC
CAGTCCGAGACCCACCCTCCCCTGACTTGGCAGAAGTGCTCCTCTGGCGGTACTTGTA
CCCAGCAGACCGGTTCCGTCGTGATCGACGCTAACTGGCGATGGACTCATGCCACCAA
CTCGTCCACCAACTGTTACGACGGTAACACTTGGTCTTCGACCCTCTGCCCCGATAACG
AGACCTGTGCTAAGAACTGCTGTCTGGACGGAGCCGCTTACGCCTCCACCTACGGAGT
CACCACTTCTGGCAACTCCCTCTCTATTGGCTTCGTCACCCAGTCCGCCCAGAAGAAC
GTGGGTGCTCGACTGTACCTCATGGCCTCTGACACCACTTACCAGGAGTTTACCCTGCT
CGGTAACGAGTTCTCTTTTGACGTGGATGTTTCGCAGCTGCCCTGCGGTCTCAACGGA
GCTCTGTACTTCGTTTCCATGGACGCCGATGGAGGCGTCTCTAAGTACCCTACTAACAC
CGCCGGCGCTAAGTACGGTACCGGATACTGCGACTCTCAGTGTCCCCGAGATCTGAAG
TTCATCAACGGCCAGGCTAACGTTGAGGGTTGGGAGCCTTCCTCTAACAACGCCAACA
CCGGTATTGGTGGACACGGATCTTGCTGTTCGGAGATGGACATCTGGGAGGCTAACTC
GATTTCCGAGGCCCTCACCCCTCACCCTTGCACCACTGTGGGCCAGGAGATCTGCGAG
GGCGACGGTTGTGGCGGTACCTACTCCGATAACCGATACGGAGGCACTTGCGACCCCG
ATGGATGTGATTGGAACCCTTACCGACTGGGCAACACCTCTTTCTACGGACCCGGCTC
GTCCTTTACTCTCGACACCACTAAGAAGCTGACTGTTGTCACCCAGTTCGAGACCTCC
GGTGCTATTAACCGATACTACGTTCAGAACGGAGTCACCTTTCAGCAGCCCAACGCCG
AGCTCGGATCTTACTCGGGCAACGAGCTGAACGACGATTACTGCACCGCCGAGGAAG
CCGAGTTCGGTGGATCTTCGTTTTCCGACAAGGGCGGTCTGACCCAGTTCAAGAAGGC
TACTTCCGGAGGCATGGTCCTCGTGATGTCTCTGTGGGACGATTACTACGCCAACATGC
TGTGGCTCGACTCTACTTACCCCACCAACGAGACTTCCTCTACCCCTGGTGCCGTCCGA
GGCTCCTGTTCTACCTCGTCCGGTGTGCCCGCTCAGGTTGAGTCGCAGTCCCCTAACG
CCAAGGTGACCTTCTCTAACATCAAGTTTGGCCCCATTGGTTCCACCGGAAACCCTTCT
GGTGGAAACCCTCCCGGCGGCAACCCTCCCGGAACCACTACCACTCGACGACCCGCT
ACCACTACCGGCTCTTCTCCTGGTCCTACCCAGTCCCACTACGGACAGTGTGGAGGCA
TTGGATACTCTGGTCCTACCGTGTGTGCTTCTGGAACTACCTGTCAGGTTCTCAACCCT
TACTACTCTCAGTGTCTGTAA

>*Pc*CBH1

ATGAAGCTTTCCACCATCCTTTTCACAGCCTGCGCTACCCTGGCTGCCGCCCTCCCTTC
CCCCATCACTCCTTCTGAGGCCGCAGTTCTCCAGAAGCGACAGCAGATCGGCACCTAC
ACTGCCGAGACCCACCCCTCCCTCTCTTGGTCGACCTGCAAGTCCGGCGGTTCTTGTA
CCACTAACTCCGGTGCCATTACCCTGGACGCTAACTGGCGATGGGTGCATGGCGTTAAC
ACCTCTACTAACTGCTACACTGGTAACACCTGGAACTCGGCCATCTGTGACACCGATGC
CTCTTGCGCTCAGGACTGTGCCCTCGACGGTGCTGATTACTCCGGCACCTACGGTATTA
CCACTTCGGGAAACTCCCTGCGACTCAACTTCGTCACCGGCTCGAACGTGGGTTCCCG
AACTTACCTGATGGCTGACAACACCCACTACCAGATCTTTGATCTGCTCAACCAGGAG
TTCACCTTTACTGTCGACGTGTCTCATCTGCCCTGCGGACTCAACGGCGCCCTGTACTT

CGTGACCATGGACGCTGATGGAGGCGTTTCCAAGTACCCTAACAACAAGGCCGGCGCT
CAGTACGGTGTCGGATACTGCGACTCTCAGTGTCCCCGAGATCTCAAGTTCATCGCCG
GCCAGGCTAACGTGGAGGGTTGGACCCCTTCCGCCAACAACGCTAACACCGGCATTG
GTAACCACGGAGCCTGCTGTGCTGAGCTGGACATCTGGGAGGCCAACTCCATTTCTGA
GGCTCTCACTCCCCATCCTTGTGATACCCCCGGTCTGTCTGTCTGCACCACTGACGCCT
GTGGTGGAACCTACTCCTCTGACCGATACGCTGGAACCTGCGACCCCGATGGCTGTGA
CTTCAACCCTTACCGACTCGGCGTCACCGACTTTTACGGCTCCGGCAAGACCGTGGAT
ACCACTAAGCCCTTCACTGTCGTGACCCAGTTTGTTACCAACGACGGAACCTCTACTG
GCTCGCTGTCCGAGATCCGACGATACTACGTCCAGAACGGTGTTGTCATTCCCCAGCCT
TCGTCCAAGATCTCTGGTATTTCGGGAAACGTGATCAACTCCGACTACTGCGCCGCTGA
GATTTCCACTTTCGGCGGTACCGCCTCTTTTAACAAGCACGGAGGCCTCACCAACATG
GCCGCTGGTATGGAGGCCGGAATGGTTCTCGTCATGTCCCTGTGGGACGATTACGCTGT
TAACATGCTGTGGCTCGACTCTACTTACCCTACCAACGCTACCGGAACTCCTGGTGCTG
CTCGAGGTACCTGTGCTACCACTTCCGGAGACCCCAAGACCGTGGAGTCTCAGTCGGG
ATCTTCGTACGTTACCTTCTCTGATATTCGAGTCGGCCCTTTCAACTCTACCTTCTCCGG
CGGCTCCTCTACCGGCGGTTCTACCACTACCACTGCCTCGCGAACCACTACCACTTCTG
CTTCGTCCACCTCGACTTCTTCGACCTCCACTGGAACCGGAGTGGCTGGACACTGGGG
ACAGTGTGGAGGCCAGGGTTGGACCGGACCTACCACTTGCGTTTCCGGCACCACTTGT
ACCGTGGTTAACCCTTACTACTCTCAGTGTCTGTAA

>*Nc*CBH1

ATGAAGCTTTCCACCATCCTTTTCACAGCCTGCGCTACCCTGGCTGCCGCCCTCCCTTC
CCCCATCACTCCTTCTGAGGCCGCAGTTCTCCAGAAGCGACAGGCCGTCTGCTCCCTG
ACCGCTGAGACTCACCCCTCCCTCAACTGGTCTAAGTGCACCTCCTCTGGTTGTACTAA
CGTTGCCGGCTCCATCACCGTCGACGCTAACTGGCGATGGACCCATATTACTTCTGGAT
CGACCAACTGTTACTCTGGCAACGAGTGGGACACCTCCCTGTGCTCTACCAACACTGA
CTGTGCCACCAAGTGCTGTGTGGATGGTGCTGAGTACTCGTCCACCTACGGCATTCAG
ACTTCCGGTAACTCCCTGTCTCTCCAGTTCGTTACCAAGGGTTCGTACTCCACTAACAT
CGGATCTCGAACCTACCTCATGAACGGCGCCGACGCTTACCAGGGATTTGAGCTGCTC
GGCAACGAGTTCACTTTTGACGTTGATGTCTCTGGAACCGGATGTGGTCTGAACGGTG
CTCTCTACTTCGTGTCTATGGACCTGGATGGCGGCAAGGCTAAGTACACCAACAACAA
GGCCGGTGCTAAGTACGGAACCGGATACTGTGACGCTCAGTGTCCCCGAGATCTCAAG
TACATCAACGGCATTGCTAACGTTGAGGGTTGGACCCCTTCTACTAACGACGCCAACG
CTGGTATCGGAGATCACGGAACCTGCTGTTCGGAGATGGACATTTGGGAGGCCAACAA
GGTCTCTACCGCTTTCACTCCCCACCCTTGCACCACTATTGAGCAGCATATGTGCGAGG
GCGACTCTTGTGGAGGCACCTACTCGGACGACCGATACGGTGGAACCTGCGACGCCGA
TGGCTGTGACTTCAACTCCTACCGAATGGGTAACACCACTTTTTACGGCGAGGGCAAG
ACCGTTGATACTTCTTCGAAGTTCACCGTCGTGACTCAGTTTATCAAGGACTCTGCCGG
AGATCTGGCTGAGATTAAGCGATTTTACGTCCAGAACGGCAAGGTCATCGAGAACTCT
CAGTCGAACGTCGACGGTGTGTCGGGAAACTCCATTACCCAGTCCTTCTGCAACGCCC
AGAAGACCGCTTTCGGTGACATCGACGATTTTAACAAGAAGGGCGGTCTGAAGCAGAT
GGGAAAGGCCCTGGCTAAGCCCATGGTGCTCGTTATGTCCATTTGGGACGATCACGCC
GCTAACATGCTGTGGCTCGACTCTACCTACCCCGTGGAGGGAGGCCCTGGTGCTTACC
GAGGAGAGTGTCCCACCACTTCTGGTGTGCCTGCCGAGGTTGAGGCCAACGCTCCCA

ACTCGAAGGTCATCTTCTCCAACATTAAGTTTGGCCCTATCGGTTCTACCTTCTCCGGC
GGCTCCTCTGGAACCCCTCCCTCCAACCCCTCGTCCTCTGTTAAGCCTGTCACCTCTAC
TGCCAAGCCCTCGTCCACCTCCACTGCTTCTAACCCCTCTGGAACCGGTGCTGCTCATT
GGGCTCAGTGTGGCGGTATTGGATTCTCCGGCCCCACCACTTGCCAGTCTCCTTACACC
TGTCAGAAGATCAACGACTACTACTCCCAGTGTGTGTAA
